# Preterm pigs for preterm birth research: reasonably feasible

**DOI:** 10.3389/fphys.2023.1189422

**Published:** 2023-07-14

**Authors:** Jing Sun, Jie Chong, Jinwei Zhang, Liangpeng Ge

**Affiliations:** ^1^ Chongqing Academy of Animal Sciences, Chongqing, China; ^2^ National Center of Technology Innovation for Pigs, Chongqing, China; ^3^ Key Laboratory of Pig Industry Sciences, Ministry of Agriculture, Chongqing, China

**Keywords:** preterm birth, gestational age, animal models, preterm pigs, postnatal development, application, pediatric diseases

## Abstract

Preterm birth will disrupt the pattern and course of organ development, which may result in morbidity and mortality of newborn infants. Large animal models are crucial resources for developing novel, credible, and effective treatments for preterm infants. This review summarizes the classification, definition, and prevalence of preterm birth, and analyzes the relationship between the predicted animal days and one human year in the most widely used animal models (mice, rats, rabbits, sheep, and pigs) for preterm birth studies. After that, the physiological characteristics of preterm pig models at different gestational ages are described in more detail, including birth weight, body temperature, brain development, cardiovascular system development, respiratory, digestive, and immune system development, kidney development, and blood constituents. Studies on postnatal development and adaptation of preterm pig models of different gestational ages will help to determine the physiological basis for survival and development of very preterm, middle preterm, and late preterm newborns, and will also aid in the study and accurate optimization of feeding conditions, diet- or drug-related interventions for preterm neonates. Finally, this review summarizes several accepted pediatric applications of preterm pig models in nutritional fortification, necrotizing enterocolitis, neonatal encephalopathy and hypothermia intervention, mechanical ventilation, and oxygen therapy for preterm infants.

## 1 Introduction

The World Health Organization (WHO) recommends that infants born before 37 completed weeks of gestation should be designated “preterm” (Ritchie and McClure, 1979), and 15 million babies are born prematurely each year (Locke and Kanekar, 2022). However, preterm infants are not the same as low birthweight babies. The 1991 United States national reference for fetal growth (Alexander et al., 1996) and the International Fetal and Newborn Growth Consortium for the 21st Century (INTERGROWTH-21) birth weight standards (Kozuki et al., 2015) are used to define or distinguish several key terms related to preterm birth (shown in Supplementary Table S1). A preterm newborn who is small for gestational age (SGA) has a birthweight below the 10th percentile for gestational age at birth while a preterm infant who is appropriate for gestational age (AGA) has a birthweight on the 10th-89th percentile at birth. Preterm SGA infants are at increased risk of neonatal mortality and morbidity (Bardin et al., 1997). However, it is crucial to be more careful when concluding whether there is a significant difference in the comprehensive neonatal morbidity between SGA preterm infants and AGA preterm infants due to the limitations of the research sample (Giapros et al., 2012; Marrs et al., 2015). In addition to birth weight, an essential measure for assessing the development of AGA preterm infants is the standard deviation score (SDS) of body length at birth and head circumference within the corrected age (CA) (Funkquist et al., 2010). Preterm infants born large for gestational age (LGA) are usually defined as > 90th percentile at birth. No obvious difference between LGA and AGA newborns in the relative risk of death and morbidity, meaning that LGA infants did not have a higher risk of morbidity and mortality than AGA infants (Ozawa et al., 2021). Sims et al. (Sims et al., 1992) also found that neither SGA nor AGA affected the risk of newborns developing disorders of the central nervous system. SGA preterm infants exhibited lower total iron reserves (Mukhopadhyay et al., 2012) than term AGA infants, but their amplitude-integrated electroencephalography (aEEG) patterns were more developed and durable than those of AGA preterm infants. These findings might be interpreted as a success for intrauterine compensation mechanisms (Benavente-Fernandez et al., 2017). SGA greatly increased the mortality rate of late preterm infants (born between 34 0/7 weeks through 36 6/7 weeks of gestation) (Sharma et al., 2021) and early term (37–38 weeks of gestation) (Pulver et al., 2009). These studies show that male preterm infants had a greater postnatal mortality rate than female preterm newborns and that even after full-term delivery, there may be gender-specific disparities in the survivability of neonates (Challis et al., 2013; Klein and Flanagan, 2016). Male preterm infants who were very preterm (<32 weeks gestation) (Barfield, 2018) and had very low birth weights (<1500 g) (Patel and Bhatia, 2017) had a considerably greater incidence of respiratory distress syndrome, bronchopulmonary dysplasia, severe intraventricular hemorrhage, and necrotizing enterocolitis (NEC) than did the female (Su et al., 2022; Su et al., 2022). While studies have investigated potential links between maternal ethnicity and neonatal morbidity, mortality, or treatment (Qureshi et al., 2013; Neggers, 2015; Askie et al., 2018), it is more likely that quality-of-life elements like housing conditions, sanitary conditions, or medical care will explain variations. Whatever the case, it is undeniable that preterm birth is the leading cause of death for children under the age of 5 years worldwide and that prematurity is associated with an increased occurrence of adult diseases, including retinopathy of prematurity (Hartnett and Penn, 2012), cardiovascular disease (Bavineni et al., 2019), risk of heart failure (Carr et al., 2017; Lewandowski et al., 2021), chronic lung disease (El Mazloum et al., 2014), and neurodevelopmental impairment (Blencowe et al., 2013), is undisputed. Preterm birth rates worldwide are on the rise, and according to WHO data from 2014, about 10% of babies are delivered prematurely globally (Blencowe et al., 2012; Chawanpaiboon et al., 2019). Preterm birth disrupts the development of fetal organs, and preterm newborns, particularly those born extremely preterm (<28 weeks gestation), often exhibit significant susceptibility to *postpartum* growth inhibition and infections.

## 2 Animal models for preterm birth

### 2.1 Literature retrieval

Finding appropriate animal models or human tissue models to mimic the physiological and pathological phenotypes of premature babies (Sangild, 2006; Pasca et al., 2019), as well as discovering and developing the cellular and molecular processes of preterm labor and birth (Gomez-Lopez et al., 2022), as well as determining the most effective therapeutic interventions for preterm infants (Scafidi et al., 2014; Gopalakrishna et al., 2019; Usuda et al., 2020; Sheller-Miller et al., 2021), remain major challenges for scientists to date. Eighteen animal species were referenced in the 1,010 reviewed articles (Nielsen et al., 2016) after Nielsen et al. investigated and analyzed the MEDLINE-indexed literature on animal models for the study of preterm birth processes from 1966 to January 2012. Among them, more than 100 literature used rats, sheep, mice, and pigs as preterm animal models, respectively, and as many as 76 literature used rabbits as animal models for preterm birth. In this study, an approximate count of the literature on these popular preterm animal models for the previous 10 years (up to 13 June 2023) was made using the literature search algorithm of the National Center for Biotechnology Information (NCBI) PubMed database. It was found that there were 452, 196, 132, 46, and 34 studies using mice, rats, sheep, pigs, and rabbits as preterm animal models, respectively. The retrieved reports of Review and Systematic Review were excluded from this data set.

The sheep model served as the foundation for substantial advances in perinatal human medicine and contributed significantly to our understanding of both healthy and abnormal fetal development (Morrison et al., 2018). The investigation of preterm birth routes and their genetic regulation, as well as novel discoveries about human cervical biology, have all been made possible by the mouse model (Murray et al., 2010; Mahendroo, 2012). Additionally, preterm mice models are frequently used to investigate how preterm birth affects NEC and the development of the brain or lungs (Spencer et al., 2021). The following reasons are the primary justifications for utilizing pig models in research on human preterm birth: First, in terms of anatomy, immune system function, brain growth patterns, spatial learning, and dietary requirements, newborn pigs and human neonates are very similar (Radlowski et al., 2014; Mudd and Dilger, 2017; Pabst, 2020); Second, a significant production goal sought after by pig farmers is a large litter size (Ward et al., 2020). However, fertility is easily restricted due to the sow’s limited uterine capacity, and some newborn pigs may be born underweight or at risk of preterm birth (Ward et al., 2020; Heras-Molina et al., 2021). Shorter gestations might result in significant preterm birth symptoms. It was discovered that gastrointestinal (GIT) motility and regulation, intestinal permeability and integrity, and inflammatory response were similarly immature in 88%–95% of preterm pigs during gestation and in 70%–90% of newborn neonates during gestation (Sangild, 2006). Ninety percent of newborn pigs with preterm delivery also have respiratory dysfunction (Caminita et al., 2015). Some neurodevelopmental deficits (delayed arousal, potential poor coordination of exploration and learning) endure even though many of the immature physical traits of preterm pigs adapt and disappear with age (Andersen et al., 2016). Therefore, preterm pig models are appropriate for research on the prenatal impacts of preterm birth, and newborn GIT maturation, as well as methods to improve preterm infants’ nutritional absorption and growth.

Animal models are essential research tools to study the occurrence and development of preterm birth and to evaluate potential interventions or treatments. Researchers should at least have a consensus on the selection of animal models, that is, there is not a “single ideal animal model” that can account for all aspects of labor, placental dysfunction, preterm birth, fetal growth disorders, etc. in humans. Therefore, it is important to comprehend the benefits, drawbacks, and distinctive features of various animal models of preterm birth. Even though we recognize the importance of utilizing animal models to study preterm birth in humans, we must realize that, as of yet, using these models to understand how humans are born has not substantially revealed the mechanisms that can be employed to avoid preterm birth (Bezold et al., 2013). After all, the regulation and mediating of human birth differ greatly from that of animal birth (Croy et al., 2009; Mitchell and Taggart, 2009). Pigs differ significantly from humans in terms of gestation length, number of offspring per gestation, placenta type, internal structure, and intervascular barrier characteristics. For instance, during conception, human uterine epithelial cells are lost, which results in an endometrial decidual response (Carter, 2021). However, pigs have epithelial placentas, which means that endometrial stromal cells did not defoliate since epithelial cells remained on the surface of the uterine cavity (Croy et al., 2009). These physiological differences lead to mechanisms that regulate delivery in pigs that may be different from those in humans, and these variations in physiological and environmental factors may limit the usefulness of pig models for simulating the timing of human delivery and preterm birth, as well as the degree to which studies using animal models can provide meaningful information about the physiological mechanisms of human pregnancy.

### 2.2 Animal days vs. one human year

**FIGURE 1:**
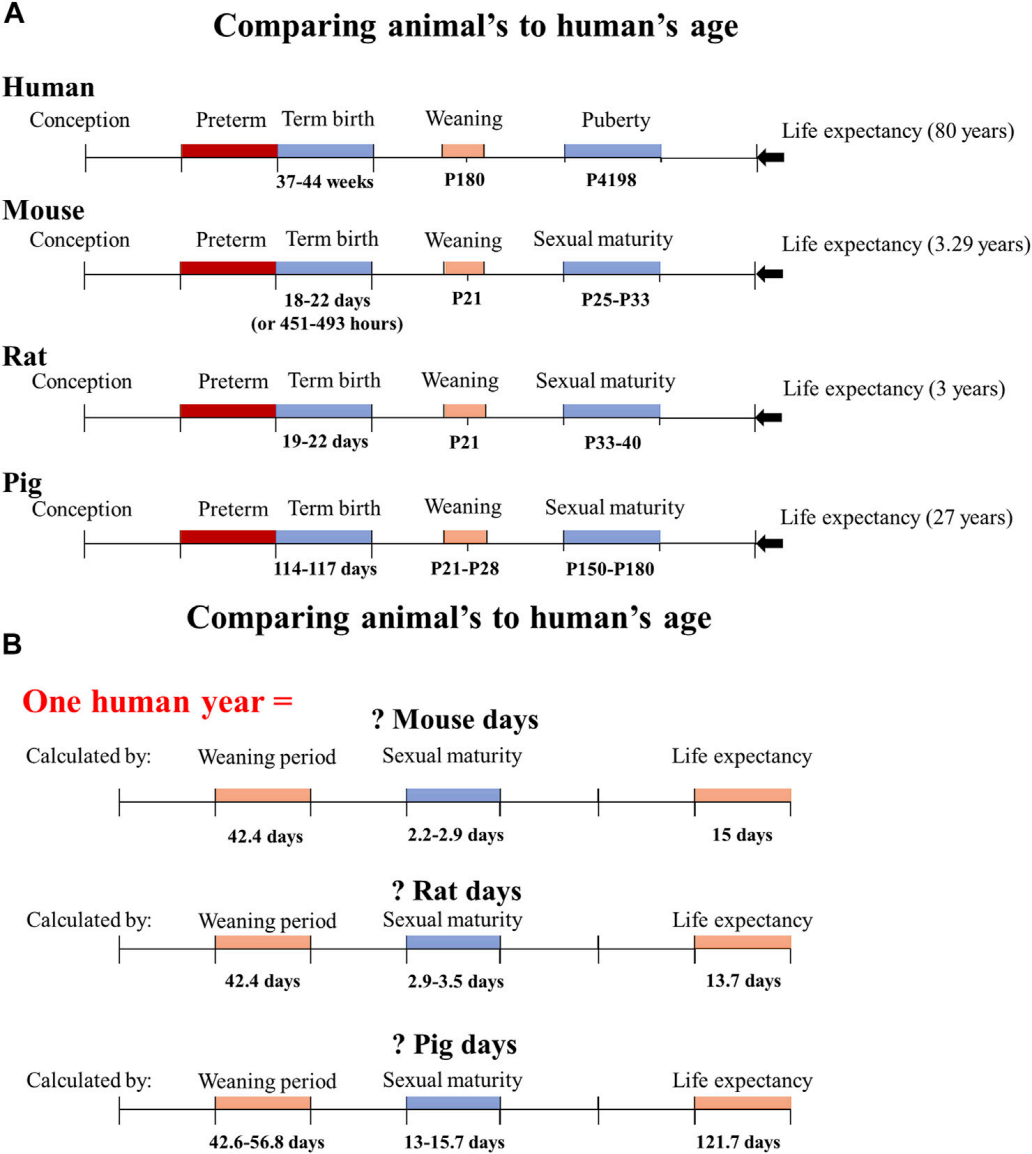
Determine how many animal days are equivalent to one human year based on the animal model’s life cycle, weaning period, and sexual maturity. **(A)** The life cycle, weaning period, sexual period, and life expectancy of humans, mice, rats, and pigs are presented based on the known literature. **(B)** The conversion of animal days to one human year based on the different growth and development stages of each animal species. P180 means the postnatal day 180.

When using animals as experimental models, researchers commonly find solutions to fundamental questions such as how an animal’s age compares to a human’s and at what age an animal is considered preterm. Therefore, establishing the relationship between the human life cycle and animal models is thus necessary (shown in Figure 1). Mice may give birth in 18–21 days (or 451–493 h) (Murray et al., 2010), reach sexual maturity in 25–33 days (Kruczek and Gruca, 1990), and live for 878–1,200 days (Schultz et al., 2020). Rats have a gestation period of 19–22 days (Grases-Pinto et al., 2019), a sexual maturity age of around 33–40 days (Quinn, 2005; Yano et al., 2020), and a life span of 2.5–3 years. Pigs have a gestation period of 114–117 days (Vanderhaeghe et al., 2011), sexual maturity period lasts for 5–6 months (Jayachandran et al., 2004) (the sexual maturity span of the Göttingen minipig is 3.7–6.5 months (Peter et al., 2016)), and they may live up to 27 years (Hoffman and Valencak, 2020). In contrast, humans have a gestation period of 37–44 weeks (Gill and Boyle, 2017; Lawson, 2021), sexual maturity (puberty) lasts for around 11.5 years, and they can live for 80 years (Sengupta, 2012). A human year is equal to 13.7 rat days, 15 mouse days, and 121.7 pig days when comparing the ages of human and animal models based on their life cycles (Quinn, 2005; Sengupta, 2013). A human year is also equal to 42.4 rat (or mouse) days, and 42.6–56.8 pig days when comparing the ages based on their weaning periods. A human year is equal to 2.9–3.5 rat days, 2.2–2.9 mouse days, and 13–15.7 pig days, depending on sexual maturity.

## 3 Physiological characteristics of preterm pig models at different gestational ages

Pig breeds have minimal influence on variations in gestational days and developmental patterns and full-term pigs are typically delivered vaginally between 114 and 117 days after conception. Studying or summarizing the onto-developmental processes and characteristics of preterm pig models may contribute to understanding the occurrence of pregnancy and preterm birth in humans, as well as research on preventive and treatment strategies. The prenatal development of fetal pigs has been the subject of extensive investigation over the past 20 years by Sangild et al. (1991); Sangild et al. (1994); Sangild et al. (1995); Sangild et al. (2013). The authors have discussed the endogenous digestive enzymes in fetal pigs at various gestational ages as well as the patterns of ontogenetic development of the adrenal glands, stomach, pancreas, and small intestine. Pig’s organ development is not linear and even the same organ’s functions may exhibit distinct developmental trajectories at birth and weaning. Pigs born prematurely had a case fatality rate that was negatively related to gestational age. Preterm pig models and preterm infants are quite similar in terms of body size, organ development, and many clinical traits, especially in the GIT maturation, which is more equivalent to human newborns (Sangild, 2006). As a result, research on organ adaptation in preterm pigs is needed to better understand the physiological mechanisms underlying preterm infant survival as well as the appropriate diet and feeding regime during the vulnerable neonatal period (Sangild et al., 2013).

Besides, sex also had an impact on the death rate and postnatal growth rate of preterm newborn pigs. Within a short period following infection, the growth rate of preterm males was lower than that of females and their mortality rate was higher than that of preterm females (Baek et al., 2021).

### 3.1 Body temperature

Pigs’ primary hair follicles development begins at 40–41 days of gestational age and lasts until 73 days of gestational age. However, secondary hair follicles disappear at birth. Pig weight gain and hair follicle length development at closely related (Meyer and Görgen, 1986). Due to their extremely poor thermoregulation and lack of hair, preterm pigs born at 91–100 days gestation have a difficult time maintaining normothermia with the aid of external heat sources. At 104–106 days of gestation, they have a hair coat but have poor thermoregulation, and need external heat sources to maintain a consistent body temperature. Pigs that are close to term at 113–114 days gestation are similar to full-term pigs in that they have well-developed skin and hair as well as sufficient thermoregulation to maintain a constant body temperature with minimal intervention (Eiby et al., 2013). Hypothermia is one of the most upsetting conditions for human newborns and has been linked to the emergence of NEC. Preterm and full-term hypothermic pigs showed considerably lower heart rates and cardiac output, and rewarming did not restore these values to return to normal (Powell et al., 1999). Hypothermia stress and hypoxia damage were used in the development of a novel NEC pig model (Cohen et al., 1991).

### 3.2 Birth weight

The breed or strain has an impact on the birth weights of both preterm and full-term pigs. The largest newborn pigs were Large white/Landrace and had an average birth weight of 697 ± 193 g (g) at day 91 of gestation, 1,095 ± 225 g at day 104 of gestation, and 1,331 ± 368 g at day 113 of gestation (Eiby et al., 2013). Preterm Yorkshire/Landrace pigs are born weighing 580 ± 150 g at 108d of gestation (Adjerid et al., 2021), whereas full-term pigs are born weighing 1,400 ± 160 g at day 115 of gestation. Averaging only 507 ± 27 g for preterm pigs born at 104 days gestation and 694 ± 56 g for term pigs even at 4 days after birth, miniature pigs had lower birth weights (Xie et al., 2022). It’s noteworthy that preterm infant pig models gained weight at a rate that was almost three times higher than that of full-term infant pigs. Birth weight, however, is not a reliable predictor of these alterations since the ability to swallow varies between preterm and full-term newborns (Adjerid et al., 2021). The difficulty to eat seems to be the main cause of the failure to thrive of extremely low-birth-weight preterm infants (Cutland et al., 2017).

### 3.3 Brain development

The brain-liver weight ratio can be used to roughly identify newborn piglets with naturally occurring asymmetric intrauterine growth restriction, offering convenient conditions for the investigation of intrauterine-impaired neonates’ pathological motivation (Eiby et al., 2013). The average ratio of brain to liver weight was 1.20 ± 0.29 when the preterm pigs were at 91–98 days of gestation and increased to 1.15 ± 0.26 at 104–106 days of gestation, while the brain to the liver weight of the near-term pigs (at days 113–114 of gestation) was 0.72 ± 0.21 (Eiby et al., 2013). Preterm birth alters the brain’s development *in utero* and may result in delayed *postpartum* neurological maturation. In comparison to full-term pigs, preterm pigs at day 106 of gestation had increased cerebral hydration, higher blood-cerebrospinal fluid permeability, higher plasma ratios of albumin and raffinose in cerebrospinal fluid (CSF), lower CSF glucose levels, and less white matter myelination (Brunse et al., 2018; Holme Nielsen et al., 2018). Preterm pigs’ neuromuscular function developed quickly after birth, and by the time they were 11 days old, the total weight of their brains and the weights of each of its areas were almost identical to those of term pigs (Holme Nielsen et al., 2018). But in preterm pigs, NEC raises the risk of brain damage and cognitive impairment. Piglets born prematurely with severe NEC lesions lost vertebral neurons in the hippocampus, had higher albumin-to-plasma and raffinose ratios in CSF, fewer CSF leukocytes, and higher levels of brain hydration, but neither brain myelination nor microglial density was affected (Brunse et al., 2018).

### 3.4 Respiratory system

Preterm pigs at 91–98 days of gestation experienced respiratory distress right away and can only partially breathe on their own at 100 days of gestation; they still require additional oxygen to survive. In comparison to near-term pigs (at day 113 of gestation), preterm pigs (<100 days gestation) had considerably higher mean arterial pressure (mmHg) and the fraction of inspired oxygen (FiO_2_), and maintenance glucocorticoid therapy dramatically increased the survival rate (Eiby et al., 2013). Preterm pigs born at 103–106 days gestation can breathe on their own and do not require ventilation. The near-term pigs (at 113–114 days of gestation) can breathe on their own in an environment with 21% oxygen (Eiby et al., 2013; Caminita et al., 2015). At 9 days after birth, preterm pigs’ alveolarization was significantly lower than that of their term counterparts (Xie et al., 2022), and they also responded to hypoxic stress less forcefully than term pigs (Eiby et al., 2014).

All mammalian newborns must be able to breathe and swallow simultaneously (German et al., 1996), however the timing of this coordination differs between full-term and preterm infants (Mayerl et al., 2019; Catchpole et al., 2020). According to a preterm pig study, preterm newborns have poor swallowing-breathing coordination (Mayerl et al., 2019). Preterm pigs’ developmental behavior is highly plastic, and by postnatal day 17, their swallowing rate (∼2 swallows/s) was comparable to that of full-term pigs. However, their respiratory rate during eating was lower than that of full-term pigs (Mayerl et al., 2019), and they ingested less milk each time (Mayerl et al., 2020). These findings demonstrate that full-term and preterm pig models have different feeding behaviors.

### 3.5 Cardiovascular system

Around 30% of newborns who are born extremely preterm (<30 weeks of gestation) have insufficient systemic blood flow, which can result in morbidity and even death. Cardiovascular health in preterm pigs was much worse than in full-term pigs. They had significantly lower baselines for cardiac output, cerebral blood flow, and myocardial contractility than full-term pigs (Eiby et al., 2016). Preterm pigs (at 97 days of gestation) had lower cardiac output and mean arterial pressure (MAPs) than full-term pigs (at 115 days of gestation) (Eiby et al., 2016), and when blood volume was removed, these parameters further decreased, along with cerebral blood flow and oxygen delivery to the brain (Eiby et al., 2018). Preterm pigs between 91 and 98 days of gestation showed low levels of β1-adrenoceptor mRNA expression in the heart, although term pigs’ mRNA expressions of the cardiac renin-angiotensin system (RAS) and II-AT1R were comparable. At 100 days of gestation, preterm pigs exhibit unstable blood pressure, a low MAP, and a low and highly variable arterial base excess (ABE). Compared to full-term newborns, the CSF-plasma albumin ratio and the raffinose ratio of preterm newborns increased by 71% and 25%, respectively. Serum glucose and CSF glucose both decreased significantly. Piglets with severe NEC lesions had higher plasma levels of C-reactive protein and interleukin-6 as well as fewer blood thrombocytes (Kim et al., 2014; Kim et al., 2015; Brunse et al., 2018; Eiby et al., 2018).

### 3.6 Immune system

Pig gestation can be divided into three biological stages. The fetal pig’s innate immune system starts to form in response to certain immunoglobulins about day 70 days of gestation, and the naïve adaptive immune system of newborn pigs is immature yet capable of responding to some pathogens (Butler et al., 2009). Pigs, sheep, horses, and cattle are under the Group III category of mammals, whereas humans and rabbits go under the Group I category, and rodents and carnivores fall under the Group II category (Butler et al., 2006; Butler et al., 2009) depending on how immunity is transferred from the mother to the fetus. During the latter stages of pregnancy and the first day *postpartum*, immunoglobulin (IgG) is preferentially transported from maternal blood to lacteal secretions, where it is absorbed by the newborn pigs and confers passive immunity (Butler et al., 2006). As a result, colostrum from the mother is the only source of IgG for newborn pigs. The newborn preterm pigs (at 106 days of gestation) displayed a delayed development of systemic immunity during the first few postnatal weeks compared to near-term and term pigs (Nguyen et al., 2016b). For instance, preterm pigs born at 106 days of gestation showed considerable growth restriction, yet these pigs did not have immune system developmental defects (Baek et al., 2019). Although the weight of the adrenal glands in preterm pigs at 113–114 days of gestation increased, their total leucocyte counts and lymphocyte counts at delivery remained below those of full-term pigs (Baek et al., 2019). Preterm pigs born after short-term prenatal exposure to the Gram-negative endotoxin (lipopolysaccharide, LPS) have higher intestinal endotoxin, neutrophil/macrophage density, and shorter villi. The piglets do, however, show physiological adaptability to preterm birth after a few days (Pan et al., 2020b). Additionally, the histological pattern of the thymus in preterm pigs did not considerably change (Lansdown, 1977). Preterm pigs may have sterile inflammatory responses in the absence of microbial stimulus, leading to a significant increase in colonic levels of IL-12/23 p40 and IFN-γ (Splichalova et al., 2018). Additionally, preterm birth raises a newborn pig’s risk of developing *Staphylococcus epidermidis* bacteremia, and both age and diet may affect systemic immune development and gut inflammation in preterm pigs in the first few days of life (Baek et al., 2020).

### 3.7 Digestive system

Immature esophageal motility is one of the crucial aspects of esophageal development in preterm infants. Preterm pigs at 105 days of gestation had trouble initiating sucking compared to full-term pigs, and their oesophageal motility per minute (#EM/min) and propagating peristaltic wave per minute (#Prop/min) were significantly lower than those of their healthy term counterparts. There was, however, no observable trend over time. Term pigs greatly enhanced the velocity of prop and double synchronous propagating waves per minute (#DSP/min). The NEC severity grade in these healthy preterm pigs was equivalent to that in term pigs (Rasch et al., 2010).

Preterm birth alters the enzyme activity involved in nutrition digestion as well as the intestinal architecture of newborn pigs. Preterm pigs are born with low intestinal weight, shorter villi, and crypts (Aunsholt et al., 2015; Pan et al., 2020a), lower levels of sucrase, maltase, and dipeptidyl peptidase IV enzymes, and much lower levels of hexose absorption capacity than their term counterparts (Aunsholt et al., 2015). They also have reduced levels of brush border enzyme activity (lactase, sucrase, and maltase) (Buddington et al., 2012). Preterm newborn pigs at 102–104 days of gestation lacked completely developed tight junction complexes in the colon and had lower lamina propria cell counts and villous heights than full-term pigs (Splichalova et al., 2018). Pigs born preterm at 104–106 days of gestation also had higher paracellular permeability to ions and lower chloride secretion in response to the secretagogue, theophylline (Sangild et al., 1997). Preterm pigs, however, acquire modest amounts of maternal IgG by placental transfer before to birth (Butler et al., 2009), comparable to full-term pigs. This physiological function is shared by both term and preterm newborn pigs. At 2 weeks old, piglets’ enterocytes have an apical canalicular system that produces enormous vacuoles in the ileum that transmit maternal active immunoglobulins from the intestinal lumen through the intestinal epithelium (Skrzypek et al., 2007). Preterm pigs born at 106 days of gestation have equivalent intestinal permeability and glucose absorption capacities to full-term pigs (at 118 days of gestation) (Pan et al., 2020a).

### 3.8 Renal development

Preterm newborns are at risk of renal insufficiency, whereas full-term neonates do not yet have mature nephrons and full function. Compared to the arteries of full-term and 4-day-old preterm pigs, the arteries of neonatal preterm pigs (within 24 h) showed greater muscle tone and lower active wall tone (Soni et al., 2015). The development of the heart, kidneys, and renin-angiotensin system (RAS) is essential for maintaining blood pressure. Even though the kidneys of female full-term pigs expressed the genes for (pro)renin receptor, renin, angiotensinogen, angiotensin-converting enzyme (ACE), ACE2, angiotensin type 1 receptor (AT_1_R), and angiotensin type 2 receptor (AT_2_R), their renal angiotensinogen mRNA levels were lower than those of male term pigs and preterm female pigs. Angiotensin II-AT1R expression in the kidneys of preterm pigs was comparable to that of full-term pigs. However, the expression level of AT2R mRNA in the kidneys of preterm pigs that were exposed to maternally administered glucocorticoid (GC) intramuscularly was higher than those of full-term pigs (Kim et al., 2015).

To measure the passivity of renal blood volume to spontaneous changes in arterial blood pressure, the renovascular reactivity index (RVx) was devised. RVx is designed as a near-infrared spectroscopy (NIRS)-based measure that can continuously monitor the moving correlation of changes in blood volume and blood pressure in the kidney. The percentage reduction in baseline flow at the RVx threshold quartile is considered the gold standard for measuring renal vascular passivity. The RVx measurement provided a superior option for the creation of clinically viable end-organs monitoring systems to assess renal perfusion of preterm neonates during shock because it was more accurate than renal laser-Doppler measurements in detecting the reduced ability of renal blood flow (Rhee et al., 2012).

### 3.9 Blood constituents

Pigs who are born prematurely are the animal models that exhibit the typical clinical symptoms of preterm newborns (such as respiratory abnormalities, hypothermia, metabolic problems, and increased risk of infection) that develop NEC on their own after ingesting infant formula after birth (Nguyen et al., 2016a). In comparison to full-term newborns, the expression of leukocytes and their innate immune receptors (CD14, TLR2, TLR4, and MD-2) in the cord blood of preterm infants was considerably lower (Tissieres et al., 2012). Therefore, measuring blood indices and NEC characteristics in preterm pigs may be used to monitor infections, assess feeding practices, and determine when the innate immune system develops and matures. Preterm pigs with NEC exhibited lower neutrophil and lymphocyte counts than preterm pigs without NEC (Pan et al., 2022), while full-term newborn pigs had greater concentrations of total blood leukocytes (2.2-fold), neutrophils (7.1-fold), erythrocytes (1.5-fold), and hemoglobin (1.4-fold) (Nguyen et al., 2016a). Fewer neutrophils and a high proportion of progenitor cells, which made up roughly 20%–30% of total leukocytes, were present in newborn preterm pigs. Analysis of blood gene expression may be a viable strategy to help find novel early indicators of NEC, according to a recent study that indicated that whole blood gene expression in preterm pigs may be influenced before clinical symptoms of NEC become severe (Pan et al., 2022).

In comparison to term newborn pigs, neonatal preterm pigs showed lower values for blood pH, pO_2_, glucose, lactate, hematocrit (HCT), cortisol, and plasma insulin-like growth factor-I (IGF-I), and a similar plasma GLP-1 level (Andersen et al., 2016; Naberhuis et al., 2019). Growth hormone and insulin-like growth factors (IGF-I and -II) promote the growth of human fetuses and infants. Erythropoietin (EPO), growth hormone, and IGFs are all expressed early in fetal development (Halvorsen and Bechensteen, 2002). It was discovered that adding exogenous long arginine (LR3) IGF-I, an analog of IGF-I, increased the growth rate of newborn pigs (Dunshea et al., 2002). Oxygen transport to human tissues is significantly influenced by erythrocyte longevity, and IGF-I and EPO work together synergistically to influence erythrocyte formation (Halvorsen and Bechensteen, 2002). Due to its similar viscosity to human blood, sheep blood has been examined for fetal circulating red blood cell (RBC) lifespan (Brace et al., 2000; Ecker et al., 2021). Pig blood is also used to model blood circulation since, in high-flow situations, it closely resembles human blood (Weng et al., 1996). Therefore, preterm pig models can be used to measure indirect indicators like circulating RBC volume or HCT, to determine whether delayed umbilical cord clamping results in RBC transfer from placenta to newborn and the potential positive or negative effects of this treatment. (Strauss et al., 2003). The relationship between HCT and circulating RBC volume in newborns with low birth weight may also be studied using a preterm pig model to measure the infants’ capacity for systemic oxygen supply and calculate their potential need for transfusions of RBCs (Mock et al., 2001). The feasibility of extracorporeal membrane oxygenation (ECMO) to support circulatory function in newborns can also be studied using the preterm pig models (Trittenwein et al., 1999; Trittenwein et al., 2001). As a result, nursing research on preterm newborns or infants with low birth weight will benefit from comparison and analysis of the key differences in blood composition, blood biochemistry, and other physiological indexes between preterm pigs and full-term pigs.

## 4 Research applications for preterm pig models

Several physiological, morphological, and metabolic traits are similar between newborn pigs and newborn humans (Darragh and Moughan, 1995; Puiman and Stoll, 2008). Preterm pigs’ immature intestines have a strong ability for metabolic immunological reprogramming, which allows them to quickly adapt to postnatal life (Pan et al., 2020a). To choose the best food supply during the crucial newborn period, studies on physiological adaptation in preterm pigs are helpful (Sangild et al., 2013; Baek et al., 2019; Ahnfeldt et al., 2020). Therefore, preterm pigs are a good model to research the effects of preterm feeding tolerance, GIT maturation, microbial colonization, immunological activation, and growth (Obelitz-Ryom et al., 2018; Kappel et al., 2020). Using preterm pig models, the researchers found that pasteurization improved the digestion of formula milk powder and decreased bacterial adhesion in the mucosa of the proximal small intestine of piglets (Navis et al., 2020). The researchers demonstrated the significant impact of environmental influences on early life development using preterm pig models. Even though preterm pigs had a high gastrointestinal tolerance to milk that was rich in oligosaccharides, intestinal maturation, and systemic immunity were not enhanced (Obelitz-Ryom et al., 2018). Using preterm pig models, the researchers also found that oligosaccharide-enriched whey with sialyllactose can stimulate brain development and improve spatial cognition in neonates (Obelitz-Ryom et al., 2019).

### 4.1 Use in nutrient fortification and NEC protection

Breast milk is the ideal first diet for infants, especially for preterm babies. However, nutrient fortification of human milk is usually necessary to maintain the proper growth and organ development of preterm newborns when breast milk supply is limited. Preterm birth is associated with a higher risk of digestive immaturity-related problems, such as nutritional indigestion and malabsorption. It has been established that enteral nutrition given soon after birth alters the structure and function of the small intestine and that lipid digestion plays a significant role in accelerating enterocyte turnover frequency and intestinal maturation in preterm pigs (Zaworski et al., 2022). This finding suggests that a preterm infant’s appropriate intestinal growth depends on the selection of a nutritional assistance support strategy. Bovine colostrum (BC) was superior to Formula-based fortifiers (FFs) in preventing intestinal dysfunction, NEC, and systemic infection in preterm pigs (at 105–106 days of gestation) (Sun et al., 2019). In particular, neonatal and diet-sensitive preterm pigs were more susceptible to NEC, sepsis, and intestinal dysfunction as a result of FFs (Sun et al., 2018). Compared to formula, BC can promote the physical growth and intestinal development of preterm pigs (Rasmussen et al., 2016). However, the fortified formula for preterm infants performs no less admirably than fortified human milk fortification in terms of enhancing short-term weight gain in low-birth-weight preterm infants. Infant formula for preterm newborns may be a preferable choice for fortification if you want to keep expenses down and prevent feed intolerance (Chinnappan et al., 2021).

NEC is the most common gastrointestinal problem in preterm neonates. It involves the breakdown of the gut barrier disruption and bacterial invasion of the mucosa, and approximately 30% of affected infants die (Fitzgibbons et al., 2009). Because preterm pigs reproduce important aspects of human NEC and also demonstrate intestinal hypoxia comparable to that before human newborn NEC (Sangild et al., 2006; Sodhi et al., 2008; Gay et al., 2011), preterm pig models have been used to study and evaluate the effects of arginine supplements and other factors in reducing the prevalence of NEC (Sangild et al., 2006; Sodhi et al., 2008; Gay et al., 2011; Robinson et al., 2018). The production of citrulline, a precursor to arginine synthesis, and systemic production of citrulline and arginine fluxes in piglets were found to be reduced by NEC, and the reduction occurred before NEC in preterm pig models. These findings imply that decreased intestinal production of citrulline during preterm birth may be a risk factor for NEC (Robinson et al., 2018). The preterm pigs are also used to identify microorganisms that are associated with NEC (Azcarate-Peril et al., 2011) or to validate any potential NEC biomarkers (Kappel et al., 2021; Pan et al., 2022). Cow’ and sow’s colostrum (Bjornvad et al., 2008) and milk formula with osteopontin (OPN) added (Moller et al., 2011) have been shown in preterm pigs’ studies to improve gastrointestinal function and protect against NEC. There has been discussion on the ideal moment to start fortification studies in preterm babies, such as whether the first enteral feeding or when the infant’s daily milk intake exceeds 100 ml/kg of body weight (Thanigainathan and Abiramalatha, 2020). The European Milk Bank Association (EMBA) also recommends “tailored fortification” to maximize nutritional intake, while “standard fortification” is the most often used protocol in neonatal intensive care units (Arslanoglu et al., 2019). The use of very preterm and early preterm pig models in the study of various nutritional fortification products for preterm newborns should be highlighted, and their effects on improving the intestinal barrier and preventing NEC in pigs should be assessed. Due to their similar immature gastrointestinal movements compared to preterm neonates (Chen et al., 2021), preterm pig models have been used in research on the laparoscopic diagnosis of NEC (Knudsen et al., 2020). This model may sensitively and precisely predict the lesion scores of NEC, which is a crucial reference value for the diagnosis of newborn NEC and the choice of the best window for surgical intervention. Intravenous antibiotics (AB) are commonly given to newborn preterm infants to treat systemic infections, not to avoid preterm birth (Mercer and Lewis, 1997). Long-term intravenous AB exposure, however, increases the risk of antibiotic resistance in the gut microbiota (Zhang et al., 2013), NEC, or even death (Cotten et al., 2009). Enteral AB treatment in formula-fed newborn preterm pigs has improved gut health and prevented the onset of NEC (Birck et al., 2016). Brunse et al. (2021) found that enteral neomycin and amoxicillin-clavulanate treatment followed by rectal fecal microbiota transplantation (FMT) failed to protect against NEC. Donors, doses, durations, and delivery techniques for FMT might influence the outcomes of NEC.

### 4.2 Use in neonatal encephalopathy (NE)

Preterm birth is at risk due to chorioamnionitis (CA), which is also associated with cognitive decline and delayed neurodevelopment. Prenatal exposure to endotoxins like LPS for a short period resulted in proteome alterations in the CSF fluid and brain of preterm pigs (at 103 days of gestation), but most of these changes disappeared within a few days (Muk et al., 2022). Prenatal exposure to an intra-amniotic dose of LPS had a long-lasting impact on preterm pigs’ renal function, as evidenced by elevated levels of renal injury indicators (leucine-rich alpha-2 glycoprotein-1, kidney injury molecule-1, neutrophil gelatinase-associated lipocalin, hypoxia-inducible factor 1-alpha, and caspase 3) in renal tissue as well as increased expression of proteins associated with innate immune activation and adaptive immunity (Muk et al., 2020). Hypoxic-ischemic encephalopathy (HIE) is a recognizable and well-defined clinical syndrome in full-term infants caused by severe or prolonged hypoxic-ischemic episodes before or before birth. Hypoxic-ischemic injury (HII), its clinical course, surveillance, and outcome in preterm infants, however, remain difficult to define, mostly due to the possibility that clinical signs in younger preterm infants may be obscured by physiological immaturity (Gopagondanahalli et al., 2016). Therapeutic hypothermia (TH) is the gold standard treatment for full-term newborns with neonatal encephalopathy (NE), which involves lowering body temperature to 33°C–34°C. The benefits of TH for neonatal survival and neurodevelopment outweigh any short-term negative effects in full-term and late preterm infants with HIE since it can reduce death (Jacobs et al., 2013). A newborn is often wrapped for 72 h during TH to cool the entire body, but this prevents the baby and parents from having physical touch throughout the procedure and results in subcutaneous fat necrosis in full-term babies (Chakkarapani, 2016). Dingley et al. (Dingley et al., 2018) successfully used the neonatal esophageal heat exchanger (NEHE) device to lower the rectal temperature of newborn full-term pigs from 38.5°C to 35°C, indicating that esophageal cooling technology may become yet another way to quickly achieve therapeutic hypothermia and become a potential clinical scheme for the treatment of NE.

The risks of hypoglycemia, respiratory distress syndrome, dehydration, and circulatory instability are increased in preterm pigs that undergo enterectomy (Aunsholt et al., 2015). Hypoglycemia is associated with increased reactive oxygen and nitrogen species, decreased cell maturation, and brain apoptosis, all of which have the potential to affect preterm infants’ brain development (Wayenberg and Pardou, 2008). Intraventricular hemorrhage (IVH) is a common complication of preterm birth. It has been correlated with the prognosis of periventricular leukomalacia (PVL), cerebral palsy, and developmental delay (Tarby and Volpe, 1982). However, the etiology and evolution of IVH are still unclear, and no effective treatment has been developed to reduce the damage of IVH (Ballabh, 2010; 2014; Dingley et al., 2018). Using newborn pig models, Tang et al. (2016) described the link between electrical impedance tomography (EIT) for monitoring periventricular damage and blood pressure fluctuations, which may be suitable for quantitative monitoring of neonatal IVH. The use of preterm pig models to advance the maturity of the continuous EIT technology may also be explored in the future, although it is currently uncertain how to ensure that the preterm pig models can withstand the injury caused by IVH modeling surgery.

### 4.3 Use in mechanical ventilation and oxygen therapy

Birth gestational age is a key marker of health; the younger the gestational age, the greater the risk of morbidity and mortality. Because newborns’ lungs have immature lung development and are more susceptible to mechanical ventilation and oxygen therapy (Clark et al., 2000), alternate *de novo* synthesis of several growth factors (GFs) in the lung (Warburton et al., 2000) is essential for the damage repair response and remodeling in the early postnatal period. The results of model experiments in preterm pigs (85–89 percent of gestation) demonstrated that airway pressure release ventilation (APRV) improved alveolar recruitment and enhanced functional residual capacity in preterm piglet development and maintenance, and the survival rate was significantly higher than that of the open mask ventilation control group. Poor ventilation strategy selection can result in lung injury (Arrindell et al., 2015). However, ventilator-related lung damage is prevalent in these preterm newborn infants because their lungs are still developing when they are born very early (less than 24 weeks gestation). The study demonstrated that mechanical ventilation, such as APRV, can enhance newborn pigs’ oxygenation and compliance and lessen their risk of lung damage (Kollisch-Singule et al., 2017). These results imply that neonatal APRV clinical application is essential. Regarding ventilator-induced lung damage, although the technology is still in its infancy, researchers have attempted to implant oxygenators into preterm pig models to explore the impact of artificial placenta technology on the hemodynamics and brain oxygenation of newborn pigs (Darby et al., 2021).

In a study of preterm pigs, it was discovered that mechanical ventilation during the early postnatal period interferes with the expression of lung growth factors, including platelet-derived growth factor-B (PDGF-B), IGF-I, keratinocyte growth factor (KGF), hepatocyte growth factor (HGF), vascular endothelial growth factor (VEGF), and transforming growth factor-beta 1 (TGF-β1) (Liu et al., 2008). Early treatment with inhaled nitric oxide (iNO) alone or in combination with surfactant improved oxygenation and mechanical ventilation efficiency in preterm pigs. This treatment approach may be an effective alternative for the management of severe hypoxemic respiratory failure in neonates (Yang et al., 2006; Qian et al., 2008).

## 5 Conclusion

This review explains preterm birth, analyzes the relationship between the days of animal models and one human year, and describes in detail the physiological characteristics of the growth and development of preterm pigs at different gestational ages. The establishment of more accurate experimental pig models can contribute to a better understanding of the growth of preterm neonates, the occurrence and development of neonatal disorders, as well as the prevention and treatment of diseases, ultimately leading to an increase in the survival and health status of newborns. Future study is still required to determine the precise early intervention or treatment for gestational age that can best benefit newborns’ short- and long-term development.
