# Supplementary material for: Preterm pigs for preterm birth research: reasonably feasible
Source: Front Physiol. 2023 Jul 14;14:1189422. doi: 10.3389/fphys.2023.1189422 (PMC10374951; doi:10.3389/fphys.2023.1189422)
Supplement: Supplementary file 1 [file Table1.docx]

**supplementary Table 1.** Preterm birth classification, definition, and a brief list of regional prevalence.

| **Classification** | **Definition** | **Regional prevalence** |
| --- | --- | --- |
| ***Based on birth weight*** | | |
| **Small-for-gestational-age (SGA) infants** | A birthweight that is below the 10^th^ percentile for gestational age ([Wilcox et al., 2021](#_ENREF_27)) | Newborns in South Asia make up 41.5% of SGA cases at birth, compared to 7% of infants in industrialized countries ([Campisi et al., 2019](#_ENREF_3)).  In California, there were 33447 SGA newborns in 2014 and 37603 in 2005 ([Ratnasiri et al., 2018](#_ENREF_21)). 12.28% of SGA newborns in Guangdong Province, China (during 2014-2019) ([He et al., 2021](#_ENREF_10)).  In Cambodia, Nepal, and the Occupied Palestinian Territory, the prevalence of SGA was as high as 18.8% ((852/4525), 17.9% (1874/10474), and 16.1% (142/884), respectively. In Japan, the prevalence of SGA newborns was as high as 16% (543/3391) ([Ota et al., 2014](#_ENREF_18)). |
| **Appropriate-for-gestational age (AGA) infants** | A birthweight that is between the 10^th^ and 89^th^ percentile for gestational age ([Marrs et al., 2015](#_ENREF_15)) | Japan had the largest percentage of extremely preterm infants (<26 gestational age) at 80.2% (5530/6896) ([Ozawa et al., 2021](#_ENREF_19)). According to a survey conducted in South India, the prevalence of preterm AGA newborns reached 75% (444/592) ([Anne et al., 2022](#_ENREF_2)). |
| **Large-for-gestational-age (LGA) infants** | A birth weight that is higher than the 90^th^ percentile for gestational age | In California (USA), 1.0% of 3974973 singleton births (during 2007-2014) were preterm-LGA births([Ratnasiri et al., 2018](#_ENREF_21)); In Japan, the incidence of LGA extremely preterm infants (<26 gestational age) was 5.2% (357/6898) ([Ozawa et al., 2021](#_ENREF_19)); About 25% of preterm infants in South Indian were LGA ([Anne et al., 2022](#_ENREF_2)). |
| **Low-birthweight infants** | A newborn weighing under 2500 grams | Babies with low birth weight are frequent in developing countries ([Adam et al., 2019](#_ENREF_1)). Up to 90% of newborns weighing 1000g died, a mortality rate that declines as birthweight rises. For newborns weighing 2001-2499g, the death rate was roughly 3%. Neonatal mortality can be dramatically decreased by providing pregnant women with good care ([Oruamabo RS and OA, 1988](#_ENREF_17)). |
| ***Based on gestational age*** | | |
| **Extremely preterm infants** | Preterm infants born before 28 weeks | Extremely preterm infants frequently experience hyperglycemia ([Ramel and Rao, 2020](#_ENREF_20)), and 6.1% of these infants (22-27 gestational age) have autism spectrum disorder (ASD) ([Crump et al., 2021](#_ENREF_6)). A high-income environment is more conducive to the survival of extremely preterm newborns, and the rate of extremely preterm births has remained reasonably stable over the previous ten years at roughly 0.73% of all births ([Rogers and Hintz, 2016](#_ENREF_23); [Diggikar et al., 2022](#_ENREF_8)). In developed countries, preterm newborns between 23-24 gestational age have a 50% chance of surviving, with girls often faring better than boys ([Glass et al., 2015](#_ENREF_9)). Extremely preterm infants delivered at night in Canada have greater mortality rates than those born during the day ([Rizzolo et al., 2021](#_ENREF_22)). Greek research found that 14.3% of preterm newborns responded well to treatment with rescue surfactant combined with nasal continuous positive airway pressure (NCPAP) administered within 20 minutes after delivery ([Tsakalidis et al., 2011](#_ENREF_25)). For extremely preterm newborns, a greater supply of prenatal corticosteroids may have better results ([Diggikar et al., 2022](#_ENREF_8)). |
| **Early preterm infants** | Babies who were born prematurely at 28 to 32 weeks gestation | Early preterm newborns had an ASD frequency of 2.6% ([Crump et al., 2021](#_ENREF_6)). Preterm newborns born at 28-32 gestational weeks have less variety of culturable bacteria in their gastrointestinal tracts than full-term infants (in France) ([Rouge et al., 2010](#_ENREF_24)). The initial hospital stay of the newborn was reduced by at least 8 days for every extra week of gestation (26-32 weeks of gestation) ([Manuck et al., 2016](#_ENREF_14)). Infants born in South Korea between 32 and 33 weeks are more likely than those born between 34 weeks to experience premature problems ([Lee et al., 2008](#_ENREF_13)). |
| **Moderate-to-late preterm infants** | Infants delivered prematurely during 32-36 weeks gestation | ASD was present in 1.9 percent of late-preterm newborns ([Crump et al., 2021](#_ENREF_6)). The cognitive, linguistic, and motor development of moderate-to-late preterm infants were less advanced at age 2 in Canada compared to the full-term control group, notably in the area of language ([Cheong et al., 2017](#_ENREF_5)). The ventilatory responses to CO_2_ were significantly higher in preterm infants born at 33-36 weeks than in infants born at 29-32 weeks, at 3-4 days, and 10-14 days after birth, but did not differ from full-term reference levels. About 60% of newborns were born with a birth weight of under 1500g, the prevalence of later preterm infants was 10%, 8-10% of life-threatening events occurred, and 1.37/1000 live births were affected by sudden infant death syndrome ([Hunt, 2006](#_ENREF_11)). |
| ***Based on gestational age and birth weight*** | | |
| **Preterm-SGA infants** | A preterm infant whose birth weight falls below the 10^th^ percentile for gestational age | Preterm-SGA births made up about 0.5% of the 3974973 singleton births in California, USA (from 2007 to 2014) ([Ratnasiri et al., 2018](#_ENREF_21)); in China, the prevalence of preterm-SGA infants was 2.17% (317/11474)([Chen et al., 2017](#_ENREF_4)); in Tanzanian, the proportion of preterm-SGA newborns was 0.3% (63/19269) ([Debere et al., 2022](#_ENREF_7)); and the SGA infants made up 46.14% of the 531 preterm infants in Ethiopian with birthweights below 2000g ([Debere et al., 2022](#_ENREF_7)). In Norway, SGA (31/365, in 1999-2000) was present in 8% of preterm newborns (<28 gestational age) ([Westby Wold et al., 2009](#_ENREF_26)). Pre-eclampsia-affected mothers had a higher risk of their babies developing SGA (26.8%) ([Jelin et al., 2012](#_ENREF_12)). |
| **Preterm-AGA infants** | An infant who was delivered prematurely and weighed between the 10^th^ and 89^th^ percentile for gestational age | Preterm-AGA births made up 5.6% of the 3974973 singleton births in California, USA (from 2007 to 2014) ([Ratnasiri et al., 2018](#_ENREF_21)); Preterm-AGA infants made up 53.86% of the 531 Ethiopian preterm infants with birthweights under 2000g ([Debere et al., 2022](#_ENREF_7)), whereas the percentage in Tanzanian was 15.5% (2989/19269) ([Muhihi et al., 2016](#_ENREF_16)). |

**References**

Adam, Z., Ameme, D.K., Nortey, P., Afari, E.A., and Kenu, E. (2019). Determinants of low birth weight in neonates born in three hospitals in Brong Ahafo region, Ghana, 2016- an unmatched case-control study. *BMC Pregnancy Childbirth* 19(1)**,** 174. doi: 10.1186/s12884-019-2315-6.

Anne, R.P., Vardhelli, V., Oleti, T.P., Murki, S., Reddy, G.M.M., Deshabhotla, S., et al. (2022). Propensity-Matched Comparison of Very Preterm Small- and Appropriate-for-Gestational-Age Neonates. *Indian J Pediatr* 89(1)**,** 59-66. doi: 10.1007/s12098-021-03878-3.

Campisi, S.C., Carbone, S.E., and Zlotkin, S. (2019). Catch-Up Growth in Full-Term Small for Gestational Age Infants: A Systematic Review. *Adv Nutr* 10(1)**,** 104-111. doi: 10.1093/advances/nmy091.

Chen, S., Zhu, R., Zhu, H., Yang, H., Gong, F., Wang, L., et al. (2017). The prevalence and risk factors of preterm small-for-gestational-age infants: a population-based retrospective cohort study in rural Chinese population. *BMC Pregnancy Childbirth* 17(1)**,** 237. doi: 10.1186/s12884-017-1412-7.

Cheong, J.L., Doyle, L.W., Burnett, A.C., Lee, K.J., Walsh, J.M., Potter, C.R., et al. (2017). Association Between Moderate and Late Preterm Birth and Neurodevelopment and Social-Emotional Development at Age 2 Years. *JAMA Pediatr* 171(4)**,** e164805. doi: 10.1001/jamapediatrics.2016.4805.

Crump, C., Sundquist, J., and Sundquist, K. (2021). Preterm or Early Term Birth and Risk of Autism. *Pediatrics* 148(3). doi: 10.1542/peds.2020-032300.

Debere, M.K., Haile Mariam, D., Ali, A., Mekasha, A., and Chan, G.J. (2022). Factors associated with small-for-gestational-age births among preterm babies born <2000 g: a multifacility cross-sectional study in Ethiopia. *BMJ Open* 12(11)**,** e064936. doi: 10.1136/bmjopen-2022-064936.

Diggikar, S., Nagesh, N.K., Kumar, N.A., and Aladangady, N. (2022). A study comparing short-term outcome in preterm infants of </=30 weeks gestation between a tertiary neonatal care unit in Bangalore, India and one in London, UK. *Paediatr Int Child Health* 42(1)**,** 5-11. doi: 10.1080/20469047.2022.2054916.

Glass, H.C., Costarino, A.T., Stayer, S.A., Brett, C.M., Cladis, F., and Davis, P.J. (2015). Outcomes for extremely premature infants. *Anesth Analg* 120(6)**,** 1337-1351. doi: 10.1213/ANE.0000000000000705.

He, H., Miao, H., Liang, Z., Zhang, Y., Jiang, W., Deng, Z., et al. (2021). Prevalence of small for gestational age infants in 21 cities in China, 2014-2019. *Sci Rep* 11(1)**,** 7500. doi: 10.1038/s41598-021-87127-9.

Hunt, C.E. (2006). Ontogeny of autonomic regulation in late preterm infants born at 34-37 weeks postmenstrual age. *Semin Perinatol* 30(2)**,** 73-76. doi: 10.1053/j.semperi.2006.02.005.

Jelin, A.C., Kaimal, A.J., Kuzniewicz, M., Little, S.E., Cheng, Y.W., and Caughey, A.B. (2012). Preterm preeclampsia: 32 to 37 weeks gestation. *J Matern Fetal Neonatal Med* 25(11)**,** 2198-2201. doi: 10.3109/14767058.2012.684110.

Lee, S.S., Kwon, H.S., and Choi, H.M. (2008). Evaluation of preterm delivery between 32-33 weeks of gestation. *J Korean Med Sci* 23(6)**,** 964-968. doi: 10.3346/jkms.2008.23.6.964.

Manuck, T.A., Rice, M.M., Bailit, J.L., Grobman, W.A., Reddy, U.M., Wapner, R.J., et al. (2016). Preterm neonatal morbidity and mortality by gestational age: a contemporary cohort. *Am J Obstet Gynecol* 215(1)**,** 103 e101-103 e114. doi: 10.1016/j.ajog.2016.01.004.

Marrs, C.C., Mendez-Figueroa, H., Hammad, I.A., and Chauhan, S.P. (2015). Differential Morbidity in Preterm Small versus Appropriate for Gestational Age: Perhaps Unverifiable. *Am J Perinatol* 32(13)**,** 1251-1256. doi: 10.1055/s-0035-1552939.

Muhihi, A., Sudfeld, C.R., Smith, E.R., Noor, R.A., Mshamu, S., Briegleb, C., et al. (2016). Risk factors for small-for-gestational-age and preterm births among 19,269 Tanzanian newborns. *BMC Pregnancy Childbirth* 16**,** 110. doi: 10.1186/s12884-016-0900-5.

Oruamabo RS, and OA, O. (1988). Mortality in infants less than 2500 grammes birthweight admitted into a special-care baby unit in Port Harcourt, Nigeria. *East Afr Med J.* 65(3)**,** 197-202.

Ota, E., Ganchimeg, T., Morisaki, N., Vogel, J.P., Pileggi, C., Ortiz-Panozo, E., et al. (2014). Risk factors and adverse perinatal outcomes among term and preterm infants born small-for-gestational-age: secondary analyses of the WHO Multi-Country Survey on Maternal and Newborn Health. *PLoS One* 9(8)**,** e105155. doi: 10.1371/journal.pone.0105155.

Ozawa, J., Tanaka, K., Kabe, K., Namba, F., and Neonatal Research Network of, J. (2021). Impact of being large-for-gestational-age on neonatal mortality and morbidities in extremely premature infants. *Pediatr Res* 90(4)**,** 910-916. doi: 10.1038/s41390-021-01375-z.

Ramel, S., and Rao, R. (2020). Hyperglycemia in Extremely Preterm Infants. *Neoreviews* 21(2)**,** e89-e97. doi: 10.1542/neo.21-2-e89.

Ratnasiri, A.W.G., Parry, S.S., Arief, V.N., DeLacy, I.H., Halliday, L.A., DiLibero, R.J., et al. (2018). Recent trends, risk factors, and disparities in low birth weight in California, 2005-2014: a retrospective study. *Matern Health Neonatol Perinatol* 4**,** 15. doi: 10.1186/s40748-018-0084-2.

Rizzolo, A., Shah, P.S., Bertelle, V., Makary, H., Ye, X.Y., Abenhaim, H.A., et al. (2021). Association of timing of birth with mortality among preterm infants born in Canada. *J Perinatol* 41(11)**,** 2597-2606. doi: 10.1038/s41372-021-01092-9.

Rogers, E.E., and Hintz, S.R. (2016). Early neurodevelopmental outcomes of extremely preterm infants. *Semin Perinatol* 40(8)**,** 497-509. doi: 10.1053/j.semperi.2016.09.002.

Rouge, C., Goldenberg, O., Ferraris, L., Berger, B., Rochat, F., Legrand, A., et al. (2010). Investigation of the intestinal microbiota in preterm infants using different methods. *Anaerobe* 16(4)**,** 362-370. doi: 10.1016/j.anaerobe.2010.06.002.

Tsakalidis, C., Kourti, M., Karagianni, P., Rallis, D., Porpodi, M., and Nikolaidis, N. (2011). Early rescue administration of surfactant and nasal continuous positive airway pressure in preterm infants <32 weeks gestation. *Indian Pediatr* 48(8)**,** 601-605. doi: 10.1007/s13312-011-0104-z.

Westby Wold, S.H., Sommerfelt, K., Reigstad, H., Ronnestad, A., Medbo, S., Farstad, T., et al. (2009). Neonatal mortality and morbidity in extremely preterm small for gestational age infants: a population based study. *Arch Dis Child Fetal Neonatal Ed* 94(5)**,** F363-367. doi: 10.1136/adc.2009.157800.

Wilcox, A.J., Cortese, M., McConnaughey, D.R., Moster, D., and Basso, O. (2021). The limits of small-for-gestational-age as a high-risk category. *Eur J Epidemiol* 36(10)**,** 985-991. doi: 10.1007/s10654-021-00810-z.
